# Plant Growth Promoting Rhizobacteria and Silica Nanoparticles Stimulate Sugar Beet Resilience to Irrigation with Saline Water in Salt-Affected Soils

**DOI:** 10.3390/plants11223117

**Published:** 2022-11-15

**Authors:** Khadiga Alharbi, Emad Hafez, Alaa El-Dien Omara, Abdelmoniem Awadalla, Yasser Nehela

**Affiliations:** 1 Department of Biology, College of Science, Princess Nourah bint Abdulrahman University, Riyadh 84428, Saudi Arabia; 2 Department of Agronomy, Faculty of Agriculture, Kafrelsheikh University, Kafr El-Sheikh 33516, Egypt; 3 Agricultural Research Center, Department of Microbiology, Soils, Water and Environment Research Institute, Giza 12112, Egypt; 4 Department of Agronomy, Faculty of Agriculture and Natural Resources, Aswan University, Aswan 81528, Egypt; 5 Department of Agricultural Botany, Faculty of Agriculture, Tanta University, Tanta 31511, Egypt

**Keywords:** oxidative stress, *Beta vulgaris*, silica nanoparticles, plant growth promoting rhizobacteria, soil salinity, irrigation with saline

## Abstract

Combined stressors (high soil salinity and saline water irrigation) severely reduce plant growth and sugar beet yield. Seed inoculation with plant growth-promoting rhizobacteria (PGPR) and/or foliar spraying with silica nanoparticles (Si-NP) is deemed one of the most promising new strategies that have the potential to inhibit abiotic stress. Herein, sugar beet (*Beta vulgaris*) plants were treated with two PGPR (*Pseudomonas koreensis* MG209738 and *Bacillus coagulans* NCAIM B.01123) and/or Si-NP, during two successive seasons 2019/2020 and 2020/2021 to examine the vital role of PGPR, Si-NP, and their combination in improving growth characteristics, and production in sugar beet plants exposed to two watering treatments (fresh water and saline water) in salt-affected soil. The results revealed that combined stressors (high soil salinity and saline water irrigation) increased ion imbalance (K^+^/Na^+^ ratio; from 1.54 ± 0.11 to 1.00 ± 0.15) and declined the relative water content (RWC; from 86.76 ± 4.70 to 74.30 ± 3.20%), relative membrane stability index (RMSI), stomatal conductance (*gs*), and chlorophyll content, which negatively affected on the crop productivity. Nevertheless, the application of combined PGPR and Si-NP decreased oxidative stress indicators (hydrogen peroxide and lipid peroxidation) and sodium ions while increasing activities of superoxide dismutase (SOD; up to 1.9-folds), catalase (CAT; up to 1.4-folds), and peroxidase (POX; up to 2.5-folds) enzymes, and potassium ions resulting in physiological processes, root yield, and sugar yield compared to non-treated controls under combined stressors (high soil salinity and saline water irrigation). It is worth mentioning that the singular application of PGPR improved root length, diameter, and yield greater than Si-NP alone and it was comparable to the combined treatment (PGPR+Si-NP). It was concluded that the combined application of PGPR and Si-NP has valuable impacts on the growth and yield of sugar beet growing under combined stressors of high soil salinity and saline water irrigation.

## 1. Introduction

Sugar beet (*Beta vulgaris* L.) is believed to be the second most imperative crop worldwide, after sugarcane, which is mainly a multi-use crop cultivated in many countries as an industrial crop for the production of sucrose [1]. In Egypt, the sugar beet crop was grown in a total area of 280.25 ha. (13.56 t ha^−1^ production) during the 2020/2021 season with an average root yield of 50.36 t ha^−1^ [2]. The sugar beet crop can grow in arable lands in addition to low-quality soils [3]. The national strategy for sustainable development for the cultivation and production of the sugar beet crop is to expand sugar beet cultivation in salt-affected soil and irrigation with low-quality water where some nutrients, particularly micro-nutrients, are deficient which are required for higher growth and productivity [4]. Despite the importance of sugar beet as a high-income crop for farmers, some of them still do not have sufficient experience to cultivate it; therefore, it is important to pay attention to this problem and develop strategies to solve it to improve growth under difficult conditions, which has a noticeable impact on increasing productivity [5].

Soil salinity and irrigation with poor-quality water are the main worldwide conundrums that severely affect sustainable agriculture, mainly in arid and semi-arid regions which decrease crop growth by more than 70% [6]. Worldwide, approximately 800 million hectares of land are estimated to be salinity- (397 Mha) or sodicity-affected (434 Mha). In addition, the utilization of irrigation with saline water in saline soils is considered a great challenge for plant growth and development [7]. The growth of plants irrigated with saline water and/or grown in salt-affected soil is negatively affected, due to oxidative stress, and nutritional discharge because of raised Na^+^ and Cl^−^ ions and soil dispersion resulting in the limiting of sustainable agricultural production [8]. One of the characteristics of the sugar crop is its tolerance to salinity [9]. The sugar beet crop has the potential to withstand soil electrical conductivity (E.C.) to 7.0 dS m^−1^. However higher soil salinity in corporations with irrigation with saline water has an injurious impact on the root, sugar yield, and juice quality [10].

Having in mind the above-mentioned facts, recently, agricultural policy has allowed several strategies such as plant growth-promoting rhizobacteria (PGPR) and nanotechnology to enhance the growth and productivity of field crops [11]. One such accepted approach is the use of plant growth regulators that farmers have implemented to be sustainable and cheaper as well as eco-friendly [12]. Plant growth-promoting rhizobacteria (PGPR) is deemed an alternative viable technology for enhancing plant growth and productivity [13]. PGPR as bio-fertilizers could promote plant development in various ways, such as nitrogen fixation, solubilization of phosphorus, or phyto-stimulators, that rely on PGPR species [14]. *Pseudomonas* and *Bacillus* are the most widely stated genera that are used as stress-tolerant PGPR [15] which can improve morpho-physiological characteristics under environmental stressors by different direct and indirect techniques such as producing exopolysaccharides (EPS) which prevent Na^+^ flux and decline its absorption in plants [16]. This is positively reflected in N_2_ fixation and phosphate solubilization by binding free phosphorus in non-legumes and improving nutrient uptake [17]. Seed inoculation with PGPR could mitigate the adverse effects of salt stress via the production of phytohormones, i.e., auxins (IAA), gibberellins (GA), cytokinins, and abscisic acid that has the potential to promote the systemic tolerance, as well as producing stress-induced ethylene [17].

An alternative sustainable technology in crop production is the use of nanoparticles as foliar spraying such as silicon nanoparticles (Si-NP). Si-NP has recently received great attention because of its potential to alleviate the adverse impacts of abiotic stresses [18]. Soil Amendment proved recently a positive effect on plant growth and development under soil salinity conditions due to making the nutrient flow available and moving faster to stomata pore sizes when applied in small concentrations [19]. Silica application could increase normal sugar beet growth by improving root growth, and morpho-physiological attributes along with sugar yield. Moreover, silicon application as nanocomposites could improve the antioxidant defense system, whilst declining oxidative stress, decrement Na^+^ uptake, and incrementing K^+^ uptake [20]. Foliar spraying with Si-NP improved the CO_2_ assimilation rate in plants by enhancing stomatal conductance (*gs*) in leaves leading to declined water loss through transpiration [21]. Consequently, we suppose that foliar spraying with Si-NP has a positive effect on plant growth and development under soil salinity conditions [22]. Little studies have focused on the physiological effect of Si-NP in sugar beet.

Having in mind the above-mentioned facts, the coupling of plant growth-promoting rhizobacteria (PGPR) and silica nanoparticles (Si-NP) has not been examined together to determine whether the motivating impacts of treatments (PGPR and Si-NP), could increase morpho-physiological processes under saline soil and irrigation with saline water. The current investigation was performed to assess the effect of singular and combination treatments of PGPR (*Pseudomonas koreensis* MG209738 and *Bacillus coagulans* NCAIM B.01123) and Si-NP on improving sugar beet physiological and biochemical attributes along with the antioxidant enzyme activity and sugar beet productivity under harsh environmental conditions.

## 2. Results

### 2.1. Seed Inoculation with PGPR and Si-NP Foliar Application Enhanced the Growth of Sugar Beet Plants under Water Salinity Stress

**Figure 1:**
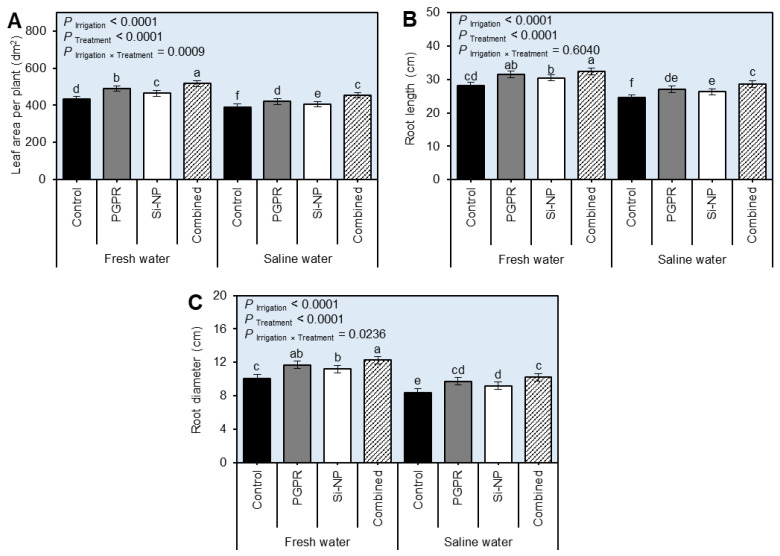
Effect of seed inoculation with PGPR and/or Si-NP foliar application on the growth of sugar beet plants grown in salt-affected soil and subjected to saline water irrigation during the 2019/2020 season. (**A**) Leaf area per plant (dm^2^), (**B**) root length (cm), and (**C**) root diameter (cm) during the 2019/2020 season, respectively. Data presented are the means ± standard deviation (mean ± SD) of three biological replicates. Different letters signify statistically significant differences between treatments according to Tukey’s HSD test (*P*_Irrigation×Treatment_ ≤ 0.05).

Although irrigation of sugar beet plants using saline water significantly (*P*_Irrigation_ < 0.0001) reduced the leaf area per plant (Figure 1A and Figure S1A), root length (Figure 1B and Figure S1B), and root diameter (Figure 1C and Figure S1C) during both 2019/2020 and 2020/2021 seasons, respectively, compared with the regularly irrigated beet plants, seed inoculation with plant growth-promoting rhizobacteria (PGPR) or silica nanoparticles (Si-NP) foliar application or their combination significantly (*P*_Treatment_ < 0.0001) enhanced all studied growth parameters (Figure 1). In both seasons, seed inoculation with PGPR individually greatly enhanced the leaf area per plant (*P*_Irrigation×Treatment_ = 0.0009 in the 2019/2020 season and *P*_Irrigation×Treatment_ = 0.0106 in 2020/2021 season) than Si-NP foliar application. However, the combined application of PGPR and Si-NP had the highest leaf area per plant, as well as root length, and root diameter during the 2019/2020 and 2020/2021 seasons when sugar beet plants were irrigated with fresh or even stressed with saline water.

### 2.2. PGPR and Si-NP Application Augmented the Water Relations and Chlorophyll Reading (SPAD) of Sugar Beet Plants under Water Salinity Stress

**Figure 2:**
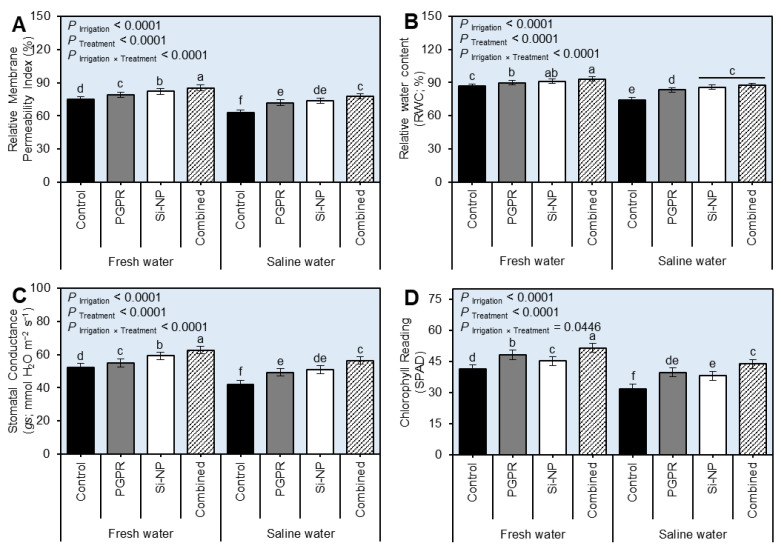
Effect of seed inoculation with PGPR and/or Si-NP foliar application on the water relations and chlorophyll reading of sugar beet plants grown in salt-affected soil and subjected to saline water irrigation during the 2019/2020 seasons. (**A**) Relative membrane permeability index (RMPI), (**B**) relative water content (RWC), (**C**) stomatal conductance (*gs*), and (**D**) chlorophyll reading (SPAD) during the 2019/2020 seasons, respectively. Data presented are the means ± standard deviation (mean ± SD) of three biological replicates. Different letters signify statistically significant differences between treatments according to Tukey’s HSD test (*P*_Irrigation×Treatment_ ≤ 0.05).

Generally, irrigation using saline water decreased (*P*_Irrigation_ < 0.0001) the relative membrane permeability index (RMPI; Figure 2A and Figure S2A), relative water content (RWC; Figure 2B and Figure S2B), and stomatal conductance (*gs*; Figure 2C and Figure S2C), as well as chlorophyll reading (SPAD; Figure 2D and Figure S2D) of beet plants compared to those irrigated with fresh water during the 2019/2020 and 2020/2021 seasons, respectively. Though, these destructive effects were significantly diminished after seed inoculation with PGPR and Si-NP foliar application, or their combination. Briefly, sugar beet plants treated with PGPR+Si-NP had the highest RMPI (*P* _Irrigation×Treatment_ < 0.0001 during both seasons), RWC (*P* _Irrigation×Treatment_ < 0.0001 during both seasons), *gs* (*P* _Irrigation×Treatment_ < 0.0001 during both seasons), SPAD reading (*P* _Irrigation×Treatment_ = 0.0446 and *P* _Irrigation×Treatment_ = 0.0011 during 2019/2020 and 2020/2021 seasons, respectively). It is worth mentioning that the combined application of PGPR and Si-NP had a better influence on both RMPI, RWC, and *gs* of beet plants than individual applications which had a greater effect on chlorophyll reading (Figure 2).

### 2.3. Seed Inoculation with PGPR and Si-NP Foliar Application Advanced the Biochemical Response of Sugar Beet Plants under Water Salinity Stress

**Figure 3:**
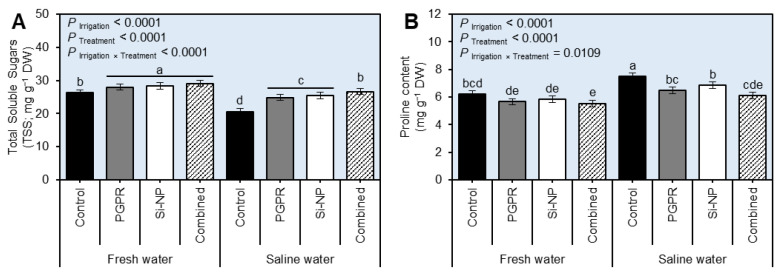
Effect of seed inoculation with PGPR and/or Si-NP foliar application on the biochemical response of sugar beet plants grown in salt-affected soil and subjected to saline water irrigation during the 2019/2020 seasons. (**A**) Total soluble sugars (TSS; mg g^−1^ DW), and (**B**) proline content (mg g^−1^ DW) during the 2019/2020 seasons, respectively. Data presented are the means ± standard deviation (mean ± SD) of three biological replicates. Different letters signify statistically significant differences between treatments according to Tukey’s HSD test (*P*_Irrigation×Treatment_ ≤ 0.05).

Irrigation of beet plants with saline water significantly reduced the leaf content of total soluble sugars (Figure 3A and Figure S3A; *P*_Irrigation_ < 0.0001) but increased the proline content (Figure 3B and Figure S3B; *P*_Irrigation_ < 0.0001), in two separate field trials during the 2019/2020 and 2020/2021 seasons. Seed inoculation with PGPR and Si-NP foliar application or their combination significantly increased the leaves content of TSS in beet plants irrigated with fresh water without significant differences between them during 2019/2020. On the other hand, the application of PGPR, Si-NP, or their combination decreased the proline content in beet leaves under both irrigation conditions (*P* _Irrigation×Treatment_ = 0.0109 and 0.0495) during 2019/2020 and 2020/2021, respectively.

### 2.4. PGPR and Si-NP Application Balanced the Leaf Content of Na^+^, K^+^, and Their Ration (K^+^/Na^+^) of Sugar Beet Plants under Water Salinity Stress

**Figure 4:**
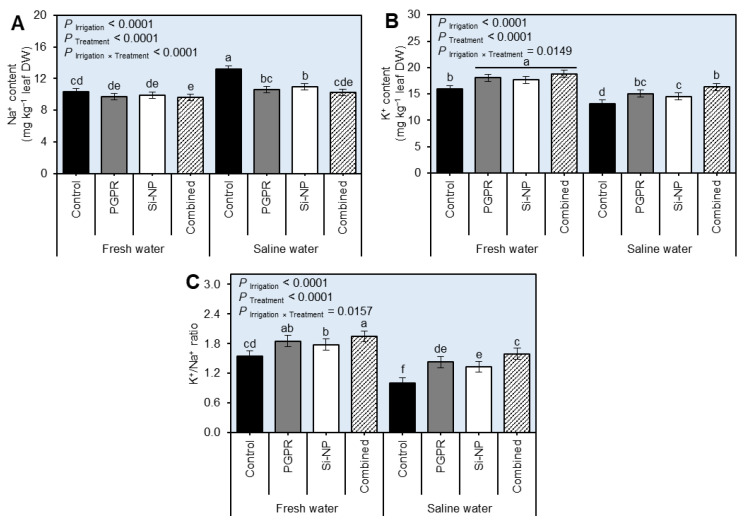
Effect of seed inoculation with PGPR and/or Si-NP foliar application on the water relations and chlorophyll reading of sugar beet plants grown in salt-affected soil and subjected to saline water irrigation during the 2019/2020 seasons. (**A**) Na^+^ content (mg kg^−1^ leaf DW), (**B**) K^+^ content (mg kg^−1^ leaf DW), and (**C**) K^+^/Na^+^ ratio during the 2019/2020 seasons, respectively. Data presented are the means ± standard deviation (mean ± SD) of three biological replicates. Different letters signify statistically significant differences between treatments according to Tukey’s HSD test (*P*_Irrigation×Treatment_ ≤ 0.05).

In general, irrigation of beet plants with saline water in the absence of PGPR and/or Si-NP markedly boosted the Na^+^ content (Figure 4A and Figure S4A during 2019/2020 and 2020/2021, respectively; *P*_Irrigation_ < 0.0001) of beet leaves but decreased the K^+^ content (Figure 4B and Figure S4B during 2019/2020 and 2020/2021, respectively; *P*_Irrigation_ < 0.0001). As a result of the disruption of Na^+^ and K^+^ content, the K^+^/Na^+^ profile was also significantly decreased in beet plants irrigated with saline water compared with fresh water-irrigated ones during both seasons (Figure 4C and Figure S4C; *P*_Irrigation_ < 0.0001). However, the application of PGPR and/or Si-NP considerably manipulated the Na^+^, K^+^, and K^+^/Na^+^ in the leaves of treated beet plants (*P*_Treatment_ < 0.0001 for the three parameters in both seasons). Briefly, seed inoculation with PGPR and Si-NP foliar application significantly decreased the Na^+^ content but increased K^+^ which resulted in a higher K^+^/Na^+^.

### 2.5. Integrated PGPR and Si-NP Application Diminished the Stress Biochemical Indicators in Sugar Beet Plants under Water Salinity Stress

**Figure 5:**
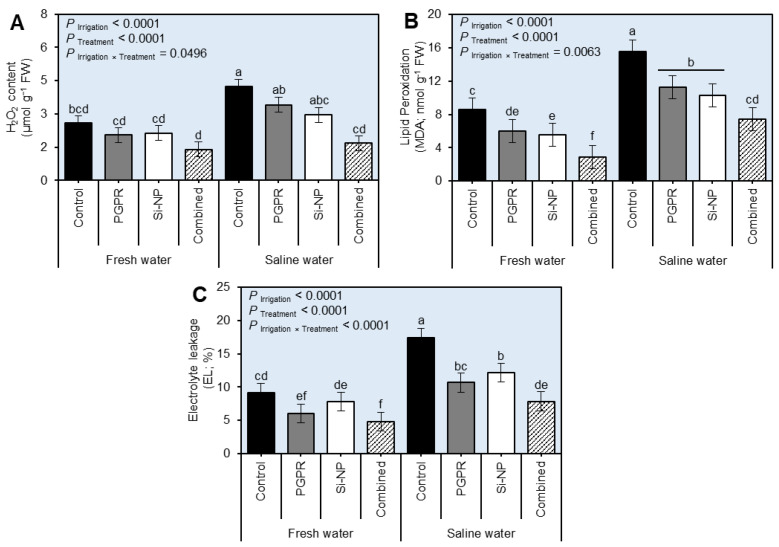
Effect of seed inoculation with PGPR and/or Si-NP foliar application on the water relations and chlorophyll reading of sugar beet plants grown in salt-affected soil and subjected to saline water irrigation during the 2019/2020 seasons. (**A**) H_2_O_2_ content (µmol g^−1^ FW), (**B**) lipid peroxidation (MDA; nmol g^−1^ FW), and (**C**) electrolyte leakage (EL; %) during the 2019/2020 seasons, respectively. Data presented are the means ± standard deviation (mean ± SD) of three biological replicates. Different letters signify statistically significant differences between treatments according to Tukey’s HSD test (*P*_Irrigation×Treatment_ ≤ 0.05).

Although irrigation of beet plants using saline water boosted the stress biochemical indicators as expressed by H_2_O_2_ content (Figure 5A and Figure S5A; *P*_Irrigation_ < 0.0001 in both seasons), lipid peroxidation (MDA; Figure 5B and Figure S5B; *P*_Irrigation_ < 0.0001 in both seasons), and electrolyte leakage (EL; Figure 5C and Figure S5C; *P*_Irrigation_ < 0.0001 in both seasons), integrated PGPR and Si-NP application notably diminished H_2_O_2_, MDA, and EL. Briefly, the application of PGPR, Si-NP, or their combination significantly reduced H_2_O_2_ content (*P*_Irrigation×Treatment_ = 0.0496 and 0.0025 in the 2019/2020 and 2020/2021 seasons, respectively) with greater impact by the combined treatment (PGPR+Si-NP). Likewise, integrated PGPR and Si-NP application significantly reduced the lipid peroxidation (*P*_Irrigation×Treatment_ = 0.0063 and 0.0076 in the 2019/2020 and 2020/2021 seasons, respectively), however, no significant differences were noticed in the MDA content between seed inoculation with PGPR and Si-NP foliar application treated plants when irrigated with saline water in the first season (2019/2020) and when plants irrigated with fresh water in the second season (2020/2021).

### 2.6. PGPR and Si-NP Application Enhanced the Antioxidant-Related Enzymatic Activity in Sugar Beet Plants under Water Salinity Stress

**Figure 6:**
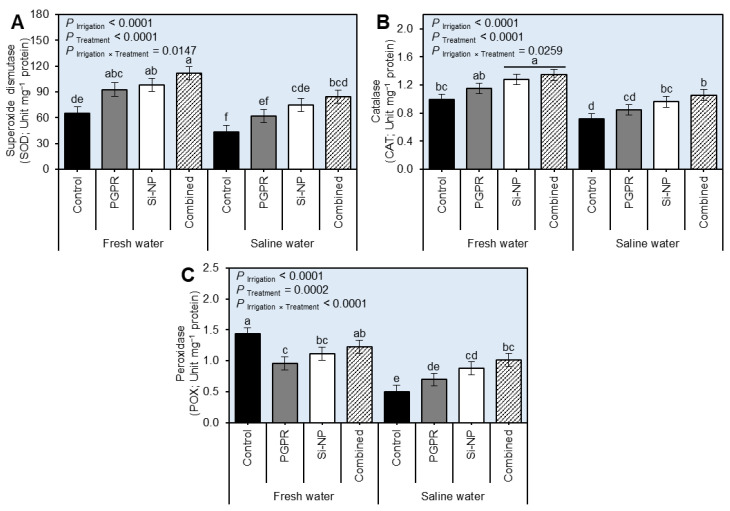
Effect of seed inoculation with PGPR and/or Si-NP foliar application on the water relations and chlorophyll reading of sugar beet plants grown in salt-affected soil and subjected to saline water irrigation during the 2019/2020 seasons. (**A**) Superoxide dismutase (SOD; Unit mg^−1^ protein), (**B**) catalase (CAT; Unit mg^−1^ protein), and (**C**) peroxidase (POX; Unit mg^−1^ protein) during the 2019/2020 seasons, respectively. Data presented are the means ± standard deviation (mean ± SD) of three biological replicates. Different letters signify statistically significant differences between treatments according to Tukey’s HSD test (*P*_Irrigation×Treatment_ ≤ 0.05).

To better understand how PGPR and Si-NP application eases the oxidative stress in saline water-irrigated beet plants, the enzymatic activities of three antioxidant-related enzymes including superoxide dismutase (SOD; Figure 6A and Figure S6A), catalase (CAT; Figure 6B and Figure S6B), and peroxidase (POX; Figure 6C and Figure S6C) were assessed in two separate field trials during the 2019/2020 and 2020/2021 seasons. Generally, in control plants (No PGRP or Si-NP), irrigation of beet plants using saline water significantly decreased the enzymatic activities of all three studied enzymes compared with those in fresh water-irrigated plants (*P*_Irrigation_ < 0.0001 for all enzymes in both seasons). However, seed inoculation with PGPR and Si-NP foliar spraying singularly or combined significantly enhanced the activities of all antioxidant enzymes including SOD (*P*_Irrigation×Treatment_ = 0.0147 and 0.0106), CAT (*P*_Irrigation×Treatment_ = 0.0259 and 0.0147), and POX (*P*_Irrigation×Treatment_ < 0.0001) in the 2019/2020 and 2020/2021 seasons, respectively, with a greater effect of combined treatment (PGPR+Si-NP).

### 2.7. Seed Inoculation with PGPR and Si-NP Foliar Application Enhanced the Yield Traits of Sugar Beet Plants under Water Salinity Stress

**Figure 7:**
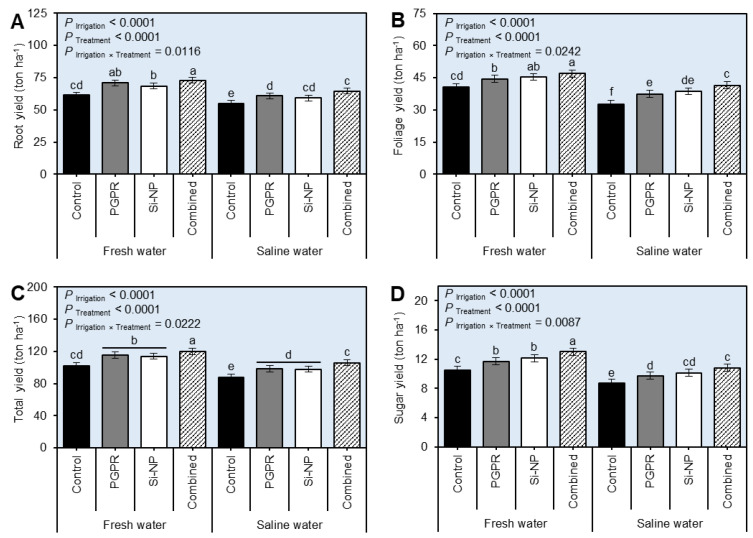
Effect of seed inoculation with PGPR and/or Si-NP foliar application on the water relations and chlorophyll reading of sugar beet plants grown in salt-affected soil and subjected to saline water irrigation during the 2019/2020 seasons. (**A**) Root yield (ton ha^−1^), (**B**) foliage yield (ton ha^−1^), (**C**) total yield (ton ha^−1^), and (**D**) sugar yield (ton ha^−1^) during the 2019/2020 seasons, respectively. Data presented are the means ± standard deviation (mean ± SD) of three biological replicates. Different letters signify statistically significant differences between treatments according to Tukey’s HSD test (*P*_Irrigation×Treatment_ ≤ 0.05).

Irrigation using saline water negatively altered the yield traits of stressed beet plants including root yield (Figure 7A and Figure S7A; *P* _Irrigation_ < 0.0001), foliage yield (Figure 7B and Figure S7B; *P*_Irrigation_ < 0.0001), total yield (Figure 7C and Figure S7C; *P*_Irrigation_ < 0.0001), and sugar yield (Figure 7D and Figure S7D; *P*_Irrigation_ < 0.0001). Nevertheless, seed inoculation with PGPR and Si-NP foliar application significantly boosted all yield traits. Briefly, the dual application of PGPR+Si-NP resulted in the highest root yield (72.93 ± 1.70 and 70.09 ± 1.60 ton ha^−1^), foliage yield (46.99 ± 1.42 and 45.62 ± 1.19 ton ha^−1^), total yield (119.92 ± 2.74 and 115.71 ± 2.79 ton ha^−1^), and sugar yield (13.04 ± 0.32 and 13.80 ± 0.33 ton ha^−1^) during 2019/2020 and 2020/2021 seasons, respectively. Similarly, integrated PGPR and Si-NP applications to saline water-irrigated beet plants resulted in higher root yield (64.48 ± 0.42 and 62.13 ± 0.39 ton ha^−1^), foliage yield (41.42 ± 0.31 and 39.67 ± 0.29 ton ha^−1^), total yield (105.90 ± 0.73 and 101.80 ± 0.68 ton ha^−1^), and sugar yield (10.83 ± 0.06 and 11.47 ± 0.06 ton ha^−1^) compared with non-treated stressed plants during 2019/2020 and 2020/2021 seasons, respectively.

## 3. Discussion

Sugar yield is assessed by the sugar biological yield that relies on the root yield and sugar content, in addition to the content of molasses-forming components (α-amino nitrogen, potassium, and sodium ions) [23]. However, soil salinity and irrigation of plants with saline water are the strongest abiotic stresses that sugar beet crops suffer in arid and semi-arid zones. They detrimentally affect plant physiological responses, sugar yield, and quality through many morph-physiological changes [24]. Accordingly, it is important to implement inexpensive plant nutrition approaches to mitigate soil salinity in lands irrigated with low-quality water in such areas worldwide. We noted that morpho-physiological characteristics and yield were severely diminished in salt-stressed plants irrigated with salt water [25]. Although, when we applied silicon nanoparticles as foliar spray or plant growth-promoting rhizobacteria (PGPR) as seed inoculation, those limiting effects were mitigated; these potential benefits were even more pronounced when applied in combination compared to untreated plants. Because these combinations make nutrients more available than in untreated plants under salt-affected soil conditions [26].

Retaining higher potassium content at the expense of the soil solution content of sodium in our experiment is the main objective for escaping from soil salinity as well as irrigation with lower quality water and obtaining higher growth of sugar beet plants. The current investigation was performed in a semi-arid area with low precipitation. Soil salinity and irrigation with saline water adversely affected nutrient availability causing an increase in the soil exchangeable sodium percentage (ESP) due to higher Na^+^ ions in the soil solution which causes the phenomenon of antagonism with potassium ions as well as Ca^2+^ and Mg^2+^ ions [27].

The application of PGPR as seed inoculum has the potential to synthesize auxins, primarily indole-3-acetic acid (IAA) which enhances cell elongation and may explain the stimulated plant growth [28]. For example, in our previous studies, *P. koreensis* MG209738 has been shown to enhance the growth and resilience to salinity stressors of several monocots including rice (*Oryza sativa*) [29] and maize (*Zea mays*) [30]. In vitro experiments showed that *P. koreensis* MG209738 produces IAA in its culture media and actively solubilizes phosphate [29]. Likewise, previously we proved that *B. coagulans* NCAIM B.01123 can enhance the physiological attributes, and productivity of wheat (*Triticum aestivum*) grown in salt-affected soil [31]. However, to the best of our knowledge, the effect of the combination of both strains (*P. koreensis* MG209738 + *B. coagulans* NCAIM B.01123) on dicot crops, such as sugar beet, has never been investigated previously. It is worth proposing that *B. coagulans* NCAIM B.01123, being a potential salinity and drought-tolerant PGPR, promoted sugar beet growth due to its 1-aminocyclopropane-1-carboxylate (ACC) deaminase activity that could decrease ethylene concentration in the stressed roots [32]. Microbial ACC-deaminase activity is associated with the biosynthesis and signaling of phytohormone ethylene by preventing the conversion of ACC into ethylene, which enhances the water-holding capacity and alleviates the harmful impact of salinity on roots [33]. We believe that seed inoculation with a mixture of *P. koreensis* MG209738 and *B. coagulans* NCAIM B.01123 may affect the phytohormonal balance within the treated plants. However, further studies are required to better understand the potential effect(s) of both strains on the phytohormonal profile of treated plants.

Moreover, foliar spraying of silica nanoparticles (Si-NP) can also stimulate the biosynthesis of plant hormones, and maintain physiological processes by alleviating oxidative damage by SODs, which mitigate salt stress [34]. It was also observed that the growth of sugar beet was maximized such as leaf area per plant with the combined use of PGPR and silicon nanoparticles that secrete polysaccharides from soil microbes that increased organic molecules in the atmospheric roots resulting in a reduction of Na^+^ ions and increased leaf area per plant, which improved soil physiochemical properties [35]. Based on the above-mentioned, it was also found that nutrient uptake and their content in the leaves increased, such as increased levels of potassium and a decrease in the content of sodium ions, which may indicate the secretion of IAA and exopolysaccharides under soil salinity in sugar beet plants subjected to irrigation with saline water [36]. So, the coupled impact of PGPR and Si-NP can increase K^+^ flux and decrease Na^+^ flux in sugar beet plants under irrigation with saline water in soil salinity. A similar finding has been described by [37].

The beneficial impact of PGPR and Si-NP on growth under salt-affected soil increase chlorophyll biosynthesis, stomatal conductance, relative water content as well as relative membrane permeability and total soluble sugars, thus stimulating the higher activities of photosynthesis and meristematic activity increasing cell division and enlargement [38]. Moreover, the heightened SPAD chlorophyll by Si-NP could also be ascribed to its potential to be an activator of many enzymes (especially SOD, CAT, and POX) involved in the detoxification of ROS (MDA and H_2_O_2_) produced under soil salinity and irrigation with saline water [39]. In addition, PGPR has the potential to solubilize inorganic forms and make them easy to uptake by plant roots in addition to activating soil microbes that improves soil fertility, especially the soil’s physical and chemical characteristics resulting in increased root yield [40].

Accumulation of proline and increased electrolyte leakage were found in saline-irrigated sugar beet plants growing in saline-affected soils. However, the application of PGPR coupled with Si-NP reduced the proline content and electrolyte leakage produced under these abiotic stresses [41]. It has been proven that seed inoculation with PGPR is capable to produce phytohormones, resulting in boosting the foliage and root yield due to reducing the proline content and electrolyte leakage [42]. Si-NP applied to the leaves remarkably compensated for proline and electrolyte leakage by directing the plant to synthesize more soluble sugars to ensure osmotic modifications and maintain plant growth [42]. Moreover, the application of PGPR along with Si-NP contains many essential nutrients which are released during their breakdown that increase the activity of antioxidant enzymes and reduces oxidative stress resulting in improved plant metabolism, reducing proline accumulation, and reducing electrolyte leakage in addition to its impact as a bio-stimulant in protecting plant cells [43].

PGPR application along with Si-NP could enhance vegetative growth including leaf area and phenotypic characteristics such as root length and root diameter through augmented cell division and enlargement due to increased nutrient and water uptake causing observed improvement in sugar beet quality characteristics and root yield along with sugar content under environmental stressors [44]. PGPR application combined with Si-NP could improve plant metabolism by synthesis of phytohormones, organic acid, phosphatases, IAA, gibberellins, and minerals and synthesis of auxins and ACC deaminase which ultimately enhance plant growth and crop yield [45].

The sugar yield is the result of the sucrose content in the juice extracted from the roots of the sugar beet. Nevertheless, the presence of impurities, i.e., Na^+^, K^+^, and α-amino nitrogen, in high amounts in beet juice has a detrimental molasses impact that declines the sugar produced from the beet pulp and therefore sugar production [46]. Si-NP combined with PGPR have maximized sugar yield, while the non-sugar components (Na^+^, K^+^, and α-amino nitrogen) were reduced under saline water irrigation conditions in saline-affected soils. These data agree with [47]. The positive impact of PGPR-conjugated Si-NP on sugar quality may be attributed to the increased uptake of potassium and silicon and decreasing the influx of Na^+^ ions into root cells as in our investigation, which has an important role in transporting photosynthetics (e.g., sucrose) from leaves to accumulate in storage root cells [48]. An additional possible reason for the decrease of K and α- amino nitrogen in beet juice is that K and α-amino nitrogen are the predominant osmotic metabolites through the vegetative phase under salinity [49]. Nevertheless, once the soluble sugars are in storage, these osmolytes correspondingly decrease in the storage root cells because it is compensated by the soluble sugars [50]. The increase in quality and sugar ratios due to the application of PGPR to the seeds coupled with Si-NP to the leaves can be ascribed to its role in reducing impurity materials like α-amino nitrogen, Na, and K and increasing the purity of the juice [51].

## 4. Materials and Methods

### 4.1. Experimental Design, Treatments, and Sampling

Two field trials were implemented in the experimental farm of Elamaar village in Sidi Salem zone, Governorate of Kafr El-Sheikh (31° 21′ 9″ N, 30° 50′ 27″ E), Egypt to estimate the effect of plant growth promoting rhizobacteria (two bacterial strains of *P. koreensis* and *B. coagulans*)) and foliar spraying with silica nanoparticles (Si-NP) as singularly and/or coupled under two irrigation treatments (irrigation with saline water and irrigation with fresh water) on growth and productivity of sugar beet plants (*Beta vulgaris* L. var. Poly Oscar; 10 kg seeds ha^−1^) grown in saline soil. Sugar beet seeds which were attained from Sugar Crops Research Institute, Agricultural Research Center, Giza, Egypt, were planted on 12 September 2019/2020 and repeated on 15 September 2020/2021 growing seasons. Seeds were planted mechanically at a rate of 2 to 3 balls per hill and then were thinned to keep 1 plant per hill pre the next irrigation. The chemical attributes of physicochemical properties of soil samples at a depth of 0–30 cm from the experimental site are shown in Table 1, whereas the chemical attributes of irrigation water from the experimental site are shown in Table 2.

The experimental layout was a strip plot with four replications per treatment. The main plots were assigned to two irrigation (fresh water vs. saline water) treatments (6 × 16 m each with a total area of 96 m^2^). The sub-plots (4 × 6 m each) were distributed for the treatments of seed inoculation with plant growth-promoting rhizobacteria (PGPR), foliar spray with Si-NP, their combination (PGPR+ Si-NP), and untreated plots (control). Each sub-plot (24 m^2^) included 7 ridges, that were 50 cm wide and 6 m long, with 20 cm apart between hills. In other words, each biological replicate contained approximately 56 independent plants. For sampling, five leaves were collected randomly at 80 days post-sowing (DPS) from each replicate (4 replicates per treatment). Collected leaves were chopped, mixed together, processed directly, or kept at −80 °C for further analyses. 

For PGPR treatment, two PGPR strains (*Pseudomonas koreensis* MG209738 and *Bacillus coagulans* NCAIM B.01123) were obtained from the Department of Agricultural Microbiology, Soils, Water and Environment Research Institute (SWERI), Agricultural Research Centre (ARC), Egypt. Briefly, 150 mL of 1 × 10^8^ CFU mL^−1^ from each culture per 250 g carrier and homogenously with sugar beet seeds pre-planting (final concentration 950 g ha^−1^). For Si-NP treatment, plants were foliar-sprayed twice with Si-NP (12.5 mg L^−1^) at 45 and 60 DPS. Si-NP spraying was attained by the Agricultural Microbiology Department, Sakha Agricultural Research Station, Kafr El-Sheikh, Egypt. The attributes of Si-NP (SiO_2_) were 260–320 m^2^ g^−1^ for specific surface area, 4–4.5 for pH, and 10 nm for diameter. Weeds were eliminated by up-rooting from the field three times through the crop growth stages. NPK fertilization was done using 200, 180, and 120 kg ha^−1^, respectively, as ammonium nitrate (33.5% N), Ca-superphosphate (15.5% P_2_O_5_), and K-sulfate (48% K_2_O).

### 4.2. Leaf Area per Plant (dm^2^)

Five plants were selected from the middle of each replicate at 80 days from sowing to measure leaf area according to Watson [52] using leaf area meter (LA-3000A, LI-COR Inc., Lincoln, NE, USA).

### 4.3. Inorganic Solutes

Five samples of leaves were detached at 80 days after sowing from each treatment, rinsed with distilled water, and dried for 72 h at 70 °C. Then finely ground plant samples were treated with HNO_3_: HClO_4_ (2:1 *v*/*v*) for 120 min at 220 °C. Leaf sodium content (Na^+^; mg kg^−1^ dry weight [DW]) and potassium content (K^+^; mg kg^−1^ DW) were determined using the atomic absorption flame spectrophotometer (Model AA-6400 F, Shimadzu Corporation, Nakagyo-ku, Kyoto, Japan) based on the method of Thomas [53].

### 4.4. Physiological Measurements 

#### 4.4.1. Chlorophyll Reading (SPAD)

Leaf samples were collected as described above to determine chlorophyll content by a SPAD meter (Model: SPAD-502, Konica Minolta, Chiyoda-ku, Tokyo, Japan), in the top expanded leaf as explained by Ling et al. [54].

#### 4.4.2. Stomatal Conductance (*gs*)

Stomatal conductance (mmol H_2_O m^−2^ s^−1^) was determined using a dynamic diffusion porometer (Delta-T AP4, Delta-T Devices Ltd., Cambridge, UK). Total leaf conductance (r_l_) is 1/r_l_ = 1/r_a_ + 1/r_b_; where the front (r_a_) and backside (r_b_) of the center of the leaf. 

#### 4.4.3. Relative Water Content (RWC)

Collected leaves, as described above, were directly weighed to measure the fresh weight (FW). Then samples were kept in distilled water for 24 h then weighed again to assess the leaf’s turgid weight (TW). Eventually, samples were dried at 70 °C for 72 h to assess the dry weight (DW). Then, the leaf’s relative water content was calculated using the method of Weatherley [55] using Equation (1):

(1) $$RWC=\left(\frac{FW-DW}{TW-DW}\right)\times 100$$

#### 4.4.4. Relative Membrane Permeability Index (RMPI)

Relative membrane permeability index (RMPI) was assessed as explained by Yang et al. [56] which was measured by a conductivity meter (TC-OMEGA, USA). Ten discs of leaves were put in test tubes including 20 mL of deionized water. The initial electrical conductivity (EC_0_) of each treatment was estimated by placing it in vortexes for five seconds. Then, the electrical conductivity (EC_1_) was measured again by placing the test tubes at 4 °C for a day. Finally, the electrical conductivity (EC_2_) was measured again by autoclaving at 120 °C for 20 min. Then, RMPI was computed using Equation (2):

(2) $$RMPI=1-\left(\frac{EC_{1}-EC_{0}}{EC_{2}-EC_{0}}\right)\times 100$$

### 4.5. Oxidative Stress Indicators

#### 4.5.1. Hydrogen Peroxide (H_2_O_2_) Content 

Approximately 500 mg of leaf samples were ground to a fine powder using liquid nitrogen and used to colorimetrically assess hydrogen peroxide (H_2_O_2_) after extraction with trichloroacetic acid (TCA: 0.1%) at 3000 rpm for 20 min. using method of Velikova, et al. [57]. H_2_O_2_ contents were assessed as µmol g^−1^ FW. 

#### 4.5.2. Lipid Peroxidation

Lipid peroxidation (MDA) was determined using 500 mg of ground leaf samples after homogenously extracted in 0.01% butyl hydroxyl toluene (BHT) and brewed at 95 °C. Thiobarbituric acid reactive substances (TBARS) were assessed according to Du and Bramlage [58]. Then, the extraction was subjected to centrifugation at 10,000× *g* for 15 min and assessed spectrophotometrically at 532 and 600 nm computed in nmol g^−1^ FW.

#### 4.5.3. Antioxidant Enzymes

About 500 mg ground leaf samples were used to assess the enzymatic activities of superoxide dismutase (SOD), catalase (CAT), and peroxidase (POX), after extraction in 5 mL of cold 100 mM K-phosphate buffer (pH 7, containing 0.5 g insoluble PVP, 0.1 mM EDTA and 2 mL L^−1^ Triton X-100), and centrifuging them for 15 min at 11,000× *g* at 4 °C. The activity of SOD (EC 1.15.1.1) was calculated by estimating the enzyme to produce 50% inhibition of the reduction of cytochrome c by superoxide caused by xanthine oxidase generated according to Beauchamp and Fridovich [59]. The specific activity of SOD is expressed as Unit mg^−1^ protein. Catalase (EC 1.11.1.6) activity was assessed by Aebi [60], and the absorbance was measured at 240 nm of a reaction mixture containing 1.9 mL H_2_O, 1.0 mL of 5.9 mM H_2_O_2_ in potassium phosphate buffer (pH 7.0), and 1.0 mL extract. The specific activity of CAT is expressed as Unit mg^−1^ protein. Peroxidase (EC 1.11.1.7) activity was assessed by the method of Vetter et al. [61] combining 100 μL enzyme extract with 2.9 mL 50 mM phosphate-citrate buffer (pH 6.5), 0.03% H_2_O_2_. At 430 nm, the change in absorbance was calculated for 5 min. The specific activity of POX is computed as Unit mg^−1^ protein.

### 4.6. Osmolytes Determination

#### 4.6.1. Electrolyte Leakage

Electrolyte leakage was assessed based on the technique of Naghashzadeh et al. [62]. Five samples of leaves (0.5 g) were selected randomly at 80 days after sowing from each treatment and were introduced into *10* mL distilled water and electrical conductivity (EC1) was computed after 12 h (A) by conductivity meter. Samples were later autoclaved for 15 min at 80 °C to assess electrical conductivity (C2). The electrolyte leakage was measured using Equation (3).

(3) $$EL=\left(\frac{C1}{C2}\right)\times 100$$

#### 4.6.2. Proline Content

Proline was extracted from 500 mg freeze-dried powdered samples with 3% sulfosalicylic acid and determined using the technique of Bates et al. [63]. Proline was calculated by the standard curve and computed in mg g^−1^ DW.

#### 4.6.3. Total Soluble Sugars (TSS)

Total soluble sugar was determined using the anthrone method as described by Sadasivam and Manickam [64]. Briefly, TSS was extracted with 80% ethanol (*v*/*v*) at 80 °C. The total soluble sugars were calculated by the glucose standard curve and stated as mg g^−1^ DW.

### 4.7. Morphology and Yield Measurements

#### 4.7.1. Root Length and Diameter

At the harvest time (at 177 days), five plants were selected randomly to assess root length (cm) which was computed by meter-scale from the point where the top was removed to the bottom of the root. Root diameter (cm) was assessed by the vernier caliper at the widest part of the root.

#### 4.7.2. Sugar Beet Yield

At the harvest time (at 177 days), five plants were selected randomly to assess foliage yield (ton ha^−1^), root yield (ton ha^−1^), and sugar yield (ton ha^−1^) in addition to sugar %. Roots and tops were detached individually, rinsed, and weighed to assess foliage yield (ton ha^−1^), and root yield (ton ha^−1^). Sugar content (%) was assessed by Saccharometer on a lead basis using the technique of Delta sugar company by AOAC method A.O.A.C [65]. Sugar yield (ton ha^−1^) = root yield (ton ha^−1^) × Sugar content (%).

### 4.8. Statistical Analysis

The analysis of variance (ANOVA) was used to test the significant differences among irrigation regimes (*p* _Irrigation_), treatments (*P*_Treatment_), and their interaction (*P*_Irrigation×Treatment_). Tukey’s honestly significant difference (HSD) test was used for post-hoc analysis based on the *p*-value of the interaction between irrigation regimes and treatments (*P* _Irrigation×Treatment_ < 0.05).

## 5. Conclusions

Previously, several plant growth-promoting rhizobacteria (PGPR) were isolated and characterized from numerous plant species such as tomato [66], as well as endophytes and PGPR were used in the biosynthesis of nanoparticles [67]. However, our knowledge about the potential role(s) of PGPR and Si-NP in stimulating sugar beet resilience to salinity stress (water or soil) was limited. Our findings from this study showed that the exposure of sugar beet plants to high soil salinity and irrigated with saline water simultaneously increase the osmotic stress, which augments the oxidative damage, weakens the selectivity, and diminishes the morpho-physiological characteristics. The attained findings displayed that the coupled use of PGPR and Si-NP positively lessened the detrimental impact of co-stressors (soil salinity and irrigation with saline water) in addition to enhanced morpho-physiological characteristics, productivity, and sugar beet quality. Future examinations are expected to affirm the attained findings on a large scale. Nevertheless, further studies are required to better understand the molecular and biochemical mechanisms behind how PGPR and/or Si-NP stimulate the plant response against abiotic stress.

## Figures and Tables

**Table 1:** Analysis of soil physiochemical properties during the 2019/2020 and 2020/2021 seasons.

Season	OM (%)	Soil Texture	EC (dSm^−1^)	FC (%)	pH	Cations (mmol_c_ L^−1^)				Anions (mmol_c_ L^−1^)		
						Na^+^	K^+^	Mg^+2^	Ca^+2^	Cl^−^	HCO_3_^−^	SO_4_^−2^
2019/2020	1.45	Clay loam	6.88	28.75	8.22	16.74	9.36	6.18	9.31	22.36	14.36	9.83
2020/2021	1.39	Clay loam	6.76	29.36	8.17	17.63	10.24	8.63	8.67	25.36	17.54	11.12

OM: organic matter; EC: electric conductivity; FC: field capacity.

**Table 2:** Characterization of irrigation water during the 2019/2020 and 2020/2021 growing seasons.

Character	Freshwater *		Saline Water	
	2019/2020	2020/2021	2019/2020	2020/2021
pH	7.65 ± 0.74	7.22 ± 0.71	6.25 ± 0.12	6.14 ± 0.10
EC (dS m^−1^)	0.65 ± 0.14	0.63 ± 0.02	5.84 ± 0.08	5.65 ± 0.11
SAR	1.58 ± 0.13	1.55 ± 0.04	7.88 ± 0.14	7.47 ± 0.12
Na^+^ (mmol_c_ L^−1^)	1.75 ± 0.12	1.87 ± 0.03	16.22 ± 1.35	16.58 ± 1.21
Cl^−^ (mmol_c_ L^−1^)	3.63 ± 0.13	3.54 ± 0.04	11.55 ± 0.75	11.69 ± 0.02
SO_4_^−^ (mmol_c_ L^−1^)	0.22 ± 0.02	0.11 ± 0.01	3.87 ± 0.41	4.18 ± 0.11
NH_4_^+^ (mmol_c_ L^−1^)	1.63 ± 0.04	1.74 ± 0.02	2.65 ± 0.03	2.25 ± 0.04
COD (mq L^−1^)	12.12 ± 0.85	11.12 ± 1.12	ND	ND
BOD (mq L^−1^)	5.63 ± 0.27	5.24 ± 0.23	ND	ND
SS (mq L^−1^)	199 ± 12.42	172 ± 13.45	19 ± 1.5	17 ± 1.6
DS (mq L^−1^)	299 ± 27	388 ± 25	1789 ± 118	1855 ± 117

COD: chemical oxygen demand; BOD: biological oxygen demand; SS: suspended solids; DS: dissolved solids; ND: not detected. * Well water at a depth of 20 m.

## Data Availability

The data that supports the findings of this study are contained within the article and available from the corresponding author upon reasonable request.

## Supplementary Materials

The following supporting information can be downloaded at: https://www.mdpi.com/article/10.3390/plants11223117/s1. Figure S1. Effect of seed inoculation with PGPR and/or Si-NP foliar application on the growth of sugar beet plants grown in salt-affected soil and subjected to saline water irrigation during the 2020/2021 season. Figure S2. Effect of seed inoculation with PGPR and/or Si-NP foliar application on the water relations and chlorophyll reading of sugar beet plants grown in salt-affected soil and subjected to saline water irrigation during the 2020/2021 seasons. Figure S3. Effect of seed inoculation with PGPR and/or Si-NP foliar application on the biochemical response of sugar beet plants grown in salt-affected soil and subjected to saline water irrigation during the 2020/2021 seasons. Figure S4. Effect of seed inoculation with PGPR and/or Si-NP foliar application on the water relations and chlorophyll reading of sugar beet plants grown in salt-affected soil and subjected to saline water irrigation during the 2020/2021 seasons. Figure S5. Effect of seed inoculation with PGPR and/or Si-NP foliar application on the water relations and chlorophyll reading of sugar beet plants grown in salt-affected soil and subjected to saline water irrigation during the 2020/2021 seasons. Figure S6. Effect of seed inoculation with PGPR and/or Si-NP foliar application on the water relations and chlorophyll reading of sugar beet plants grown in salt-affected soil and subjected to saline water irrigation during the 2020/2021 seasons. Figure S7. Effect of seed inoculation with PGPR and/or Si-NP foliar application on the water relations and chlorophyll reading of sugar beet plants grown in salt-affected soil and subjected to saline water irrigation during the 2020/2021 seasons.
