# Supplementary material for: Plant Growth Promoting Rhizobacteria and Silica Nanoparticles Stimulate Sugar Beet Resilience to Irrigation with Saline Water in Salt-Affected Soils
Source: Plants (Basel). 2022 Nov 15;11(22):3117. doi: 10.3390/plants11223117 (PMC9694940; doi:10.3390/plants11223117)
Supplement: Supplementary file 1 [file plants-11-03117-s001.zip › plants-1972434-supplementary.pdf]

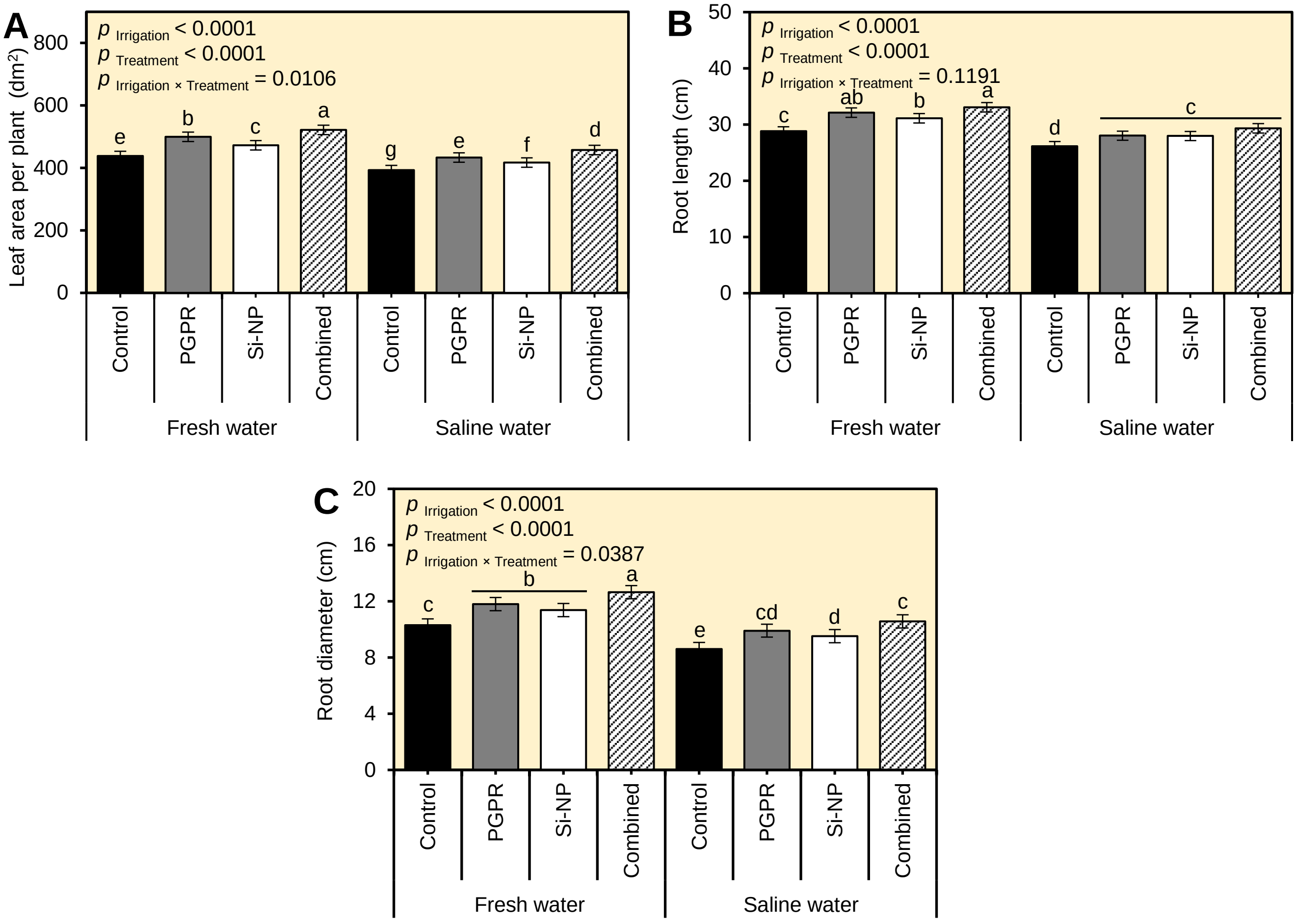


**Figure S1.** Effect of seed inoculation with PGPR and/or Si-NP foliar application on the growth of sugar beet plants grown in salt-affected soil and subjected to saline water irrigation during the 2020/2021 season. (**A**) Leaf area per plant ($dm^2$), (**B**) Root length (cm), and (**C**) Root diameter (cm) during the 2020/2021 season, respectively. Data presented are the means ± standard deviation (mean±SD) of three biological replicates. Different letters signify statistically significant differences between treatments according to Tukey's HSD test ($p_{\text{Irrigation × Treatment}} \leq 0.05$).

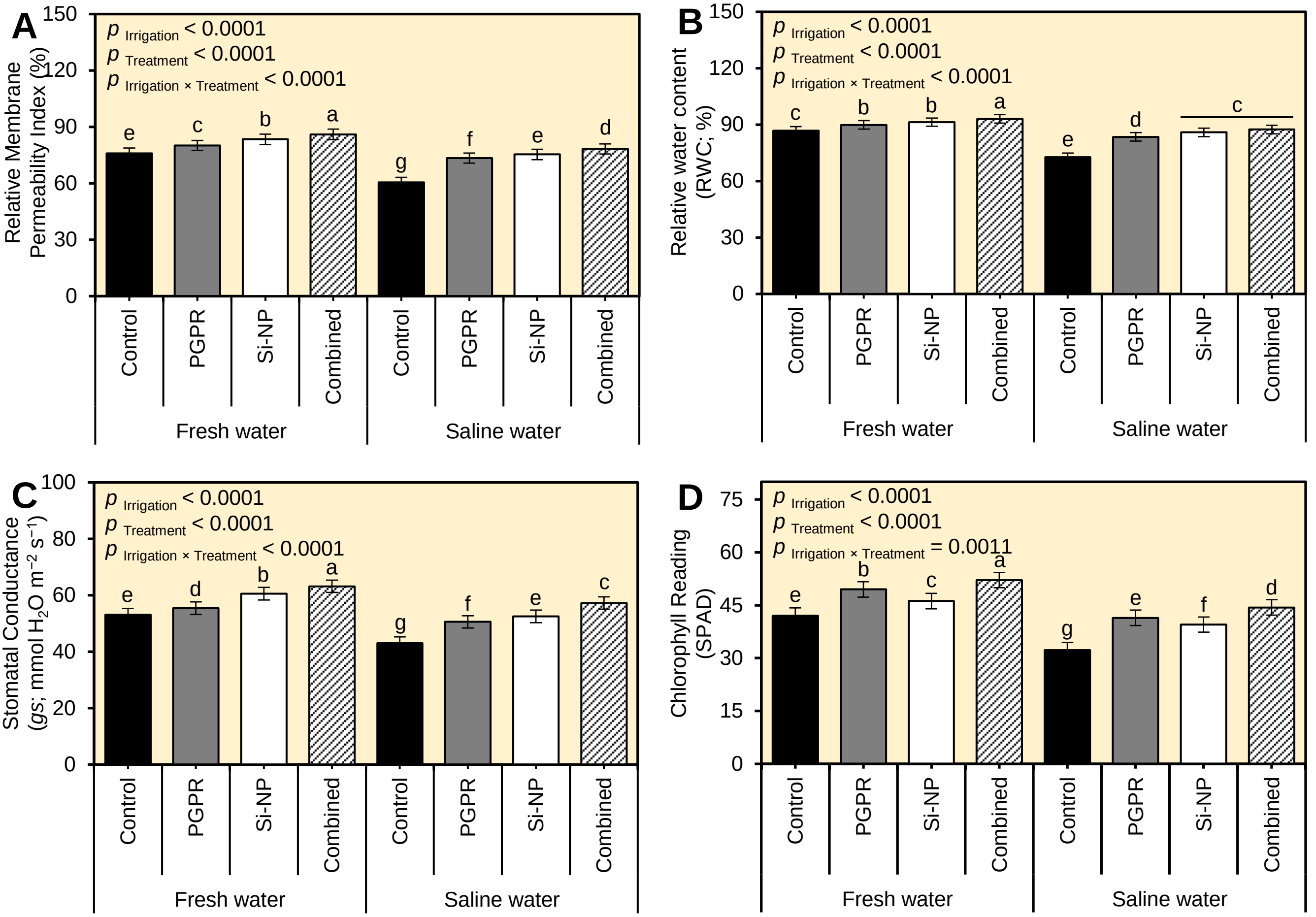


**Figure S2.** Effect of seed inoculation with PGPR and/or Si-NP foliar application on the water relations and chlorophyll reading of sugar beet plants grown in salt-affected soil and subjected to saline water irrigation during the 2020/2021 seasons. (**A**) Relative membrane permeability index (RMPI), (**B**) relative water content (RWC), (**C**) stomatal conductance (*gs*), and (**D**) chlorophyll reading (SPAD) during 2020/2021 seasons, respectively. Data presented are the means ± standard deviation (mean±SD) of three biological replicates. Different letters signify statistically significant differences between treatments according to Tukey's HSD test ($p_{\text{Irrigation} \times \text{Treatment}} \leq 0.05$).

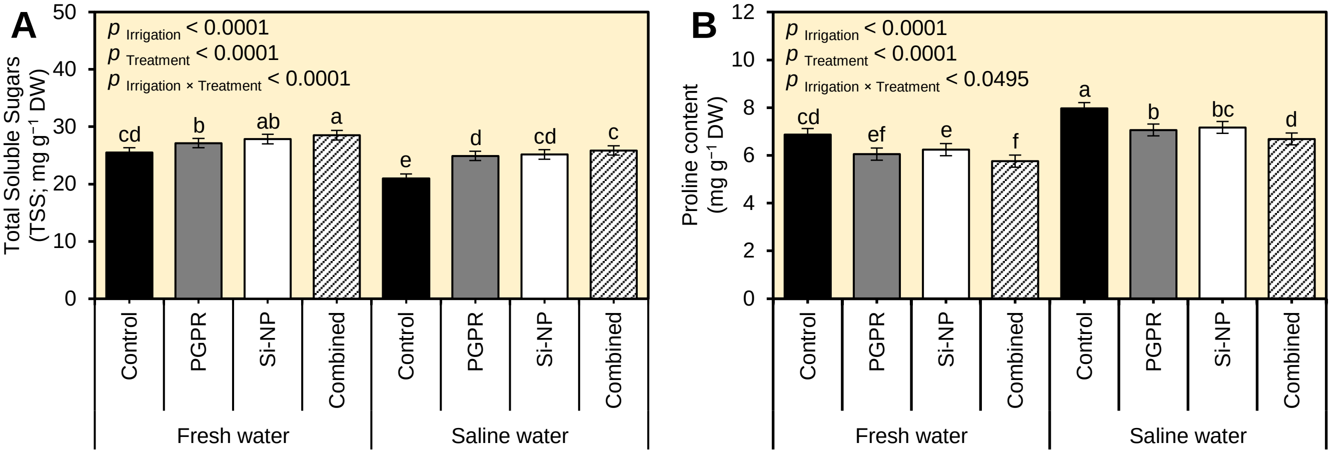


**Figure S3.** Effect of seed inoculation with PGPR and/or Si-NP foliar application on the biochemical response of sugar beet plants grown in salt-affected soil and subjected to saline water irrigation during the 2020/2021 seasons. (**A**) Total soluble sugars (TSS; mg $g^{-1}$ DW), and (**B**) Proline content (mg $g^{-1}$ DW) during the 2020/2021 seasons, respectively. Data presented are the means ± standard deviation (mean±SD) of three biological replicates. Different letters signify statistically significant differences between treatments according to Tukey's HSD test ($p_{\text{Irrigation} \times \text{Treatment}} \leq 0.05$).

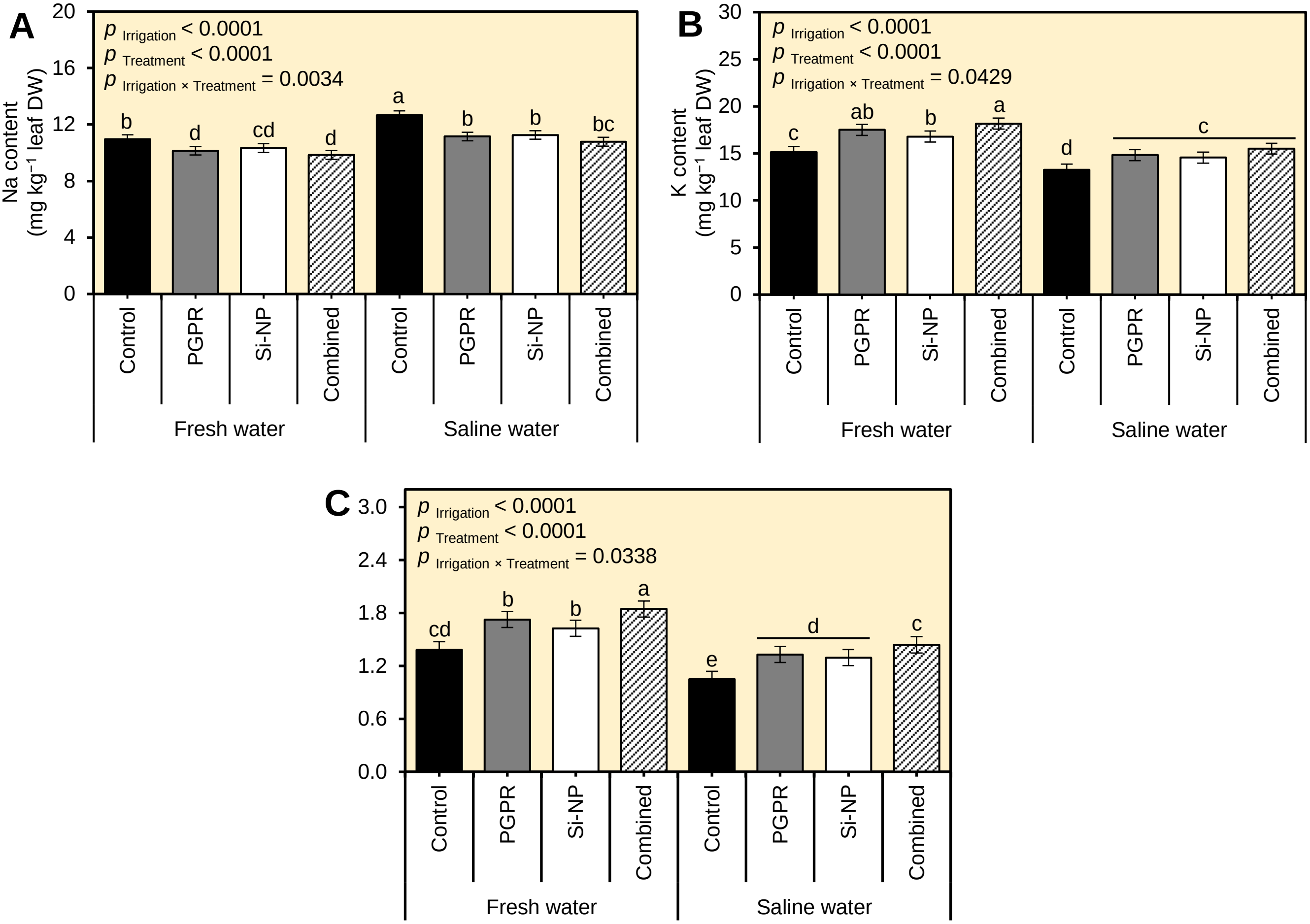


**Figure S4.** Effect of seed inoculation with PGPR and/or Si-NP foliar application on the water relations and chlorophyll reading of sugar beet plants grown in salt-affected soil and subjected to saline water irrigation during the 2020/2021 seasons. (**A**) $Na^+$ content (mg $kg^{-1}$ leaf DW), (**B**) $K^+$ content (mg $kg^{-1}$ leaf DW), and (**C**) $K^+/Na^+$ ratio during 2020/2021 seasons, respectively. Data presented are the means ± standard deviation (mean±SD) of three biological replicates. Different letters signify statistically significant differences between treatments according to Tukey's HSD test ($p_{\text{Irrigation} \times \text{Treatment}} \leq 0.05$).

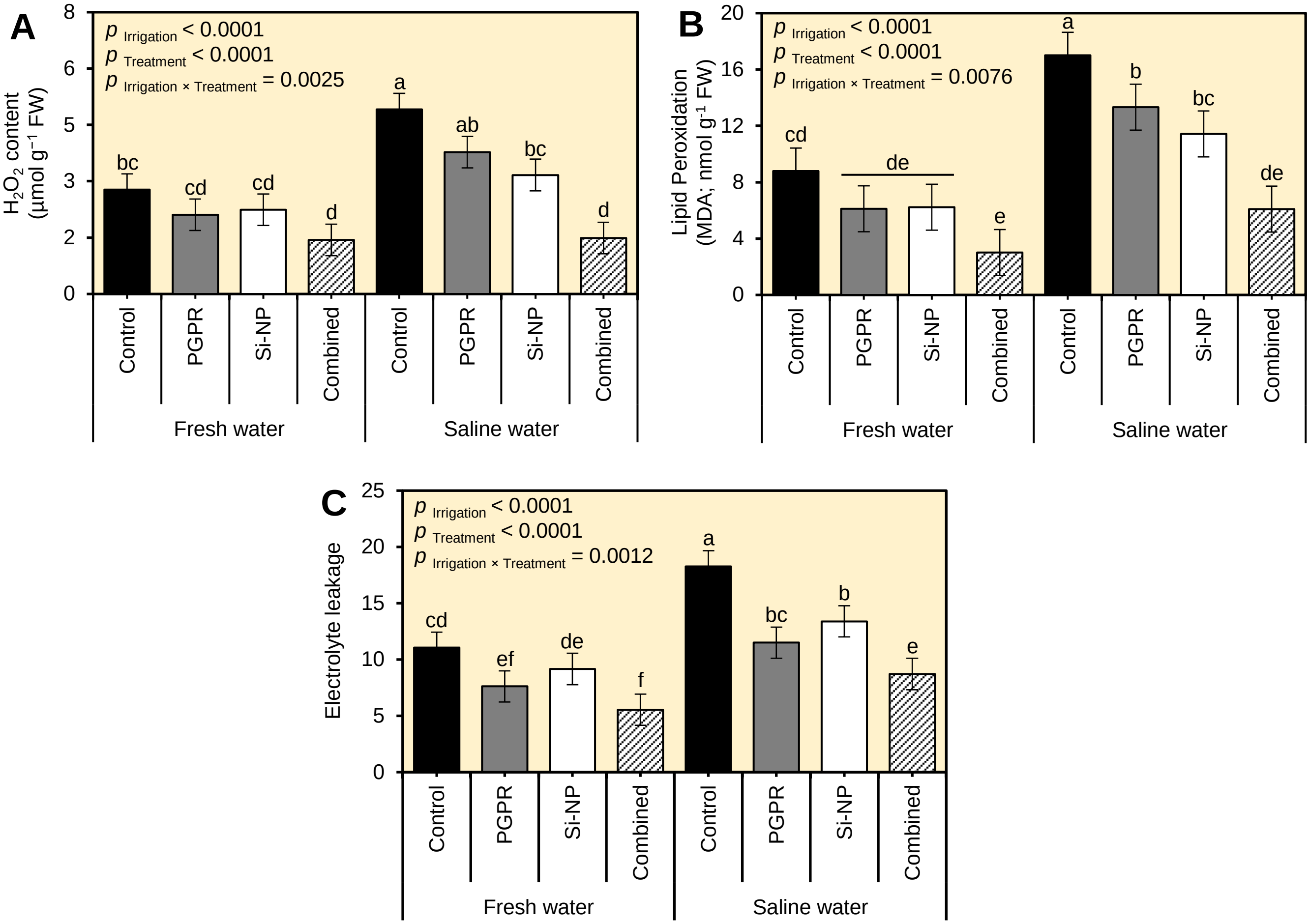


**Figure S5.** Effect of seed inoculation with PGPR and/or Si-NP foliar application on the water relations and chlorophyll reading of sugar beet plants grown in salt-affected soil and subjected to saline water irrigation during the 2020/2021 seasons. (**A**) $H_2O_2$ content (μmol $g^{-1}$ FW), (**B**) Lipid Peroxidation (MDA; nmol $g^{-1}$ FW), and (**C**) Electrolyte leakage (EL; %) during the 2020/2021 seasons, respectively. Data presented are the means ± standard deviation (mean±SD) of three biological replicates. Different letters signify statistically significant differences between treatments according to Tukey's HSD test ($p_{\text{Irrigation × Treatment}} \leq 0.05$).

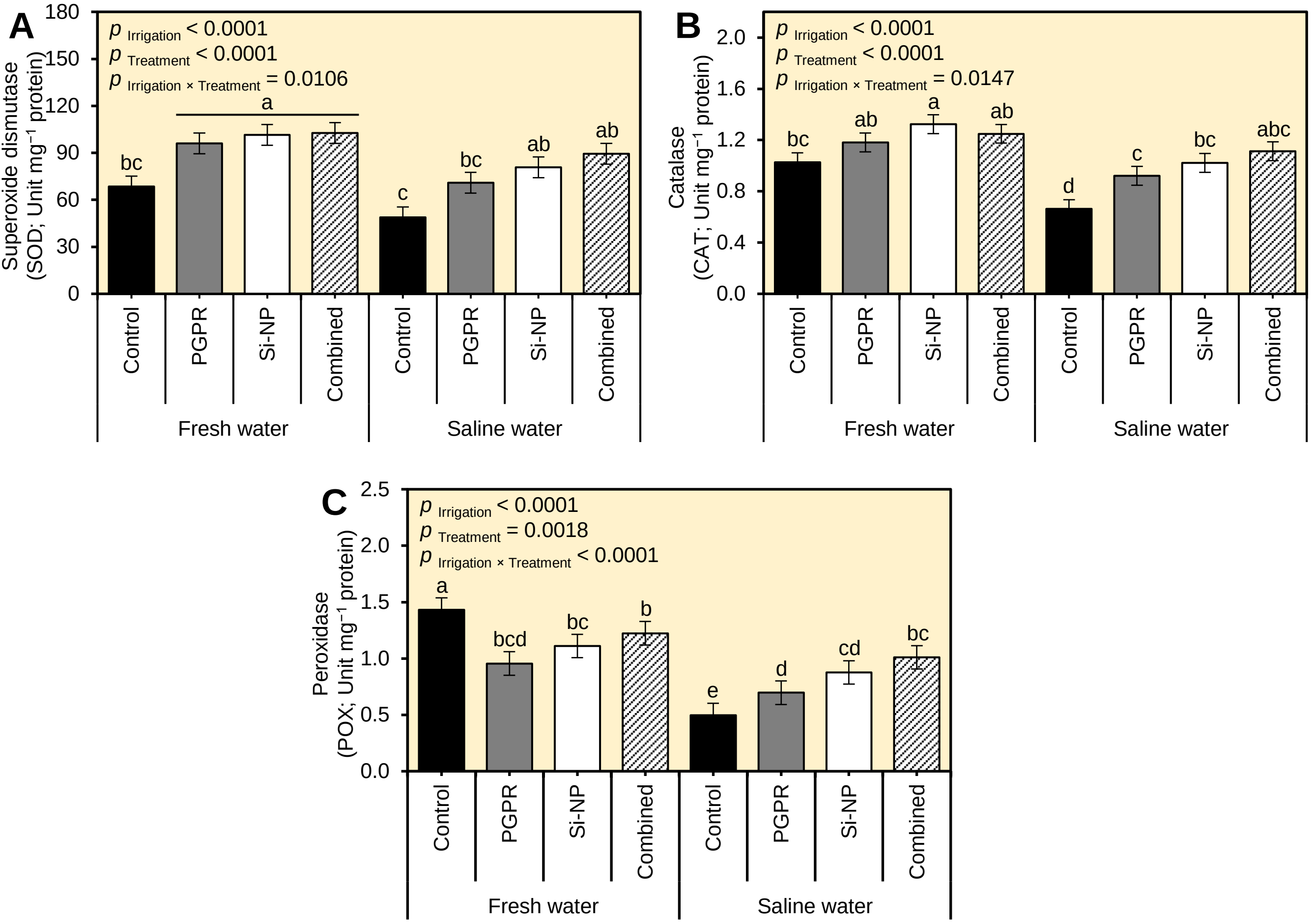


**Figure S6.** Effect of seed inoculation with PGPR and/or Si-NP foliar application on the water relations and chlorophyll reading of sugar beet plants grown in salt-affected soil and subjected to saline water irrigation during the 2020/2021 seasons. (**A**) Superoxide dismutase (SOD; Unit $mg^{-1}$ protein), (**B**) Catalase (CAT; Unit $mg^{-1}$ protein), and (**C**) Peroxidase (POX; Unit $mg^{-1}$ protein) during the 2020/2021 seasons, respectively. Data presented are the means ± standard deviation (mean±SD) of three biological replicates. Different letters signify statistically significant differences between treatments according to Tukey's HSD test ($p_{\text{Irrigation × Treatment}} \leq 0.05$).

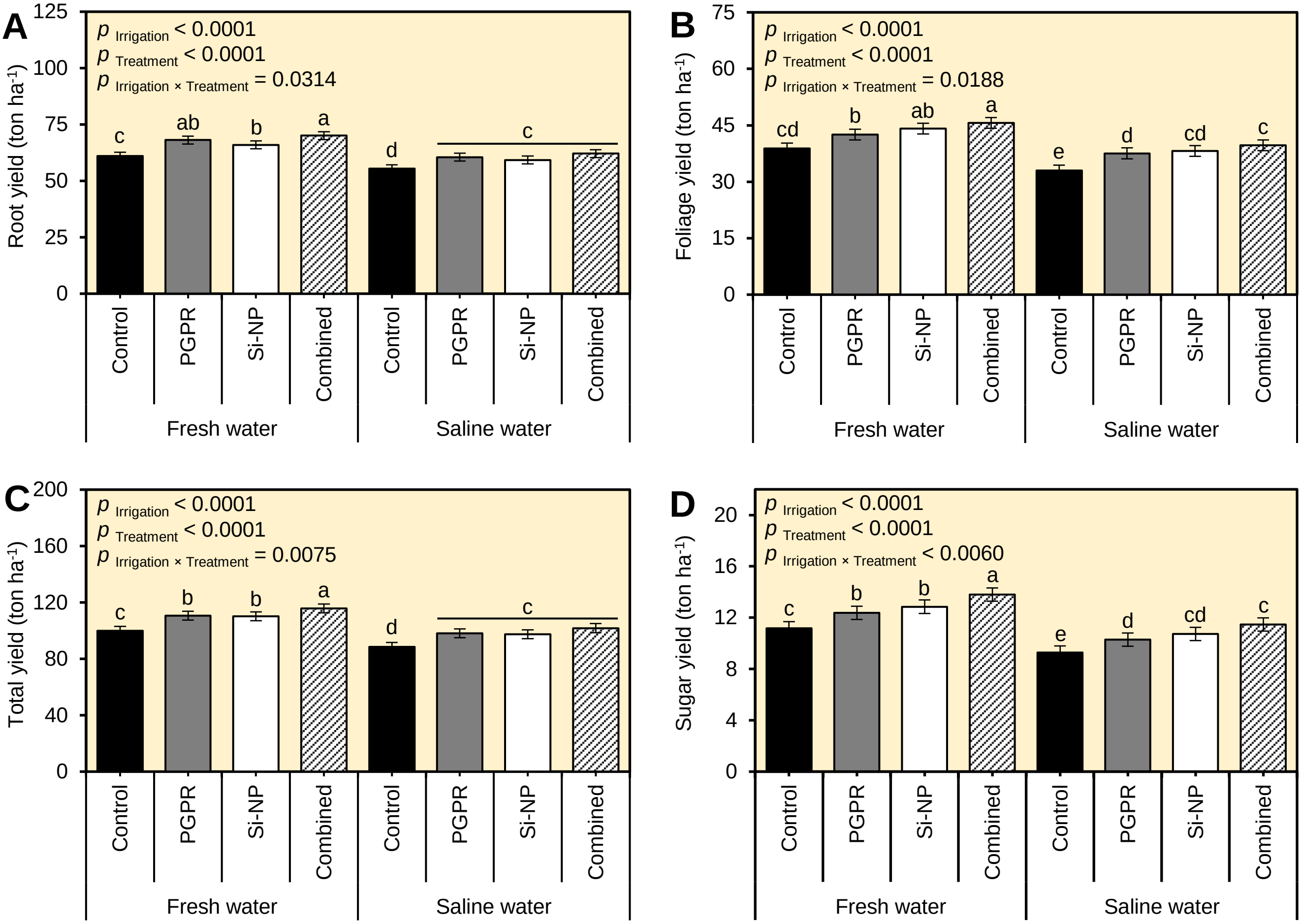


**Figure S7.** Effect of seed inoculation with PGPR and/or Si-NP foliar application on the water relations and chlorophyll reading of sugar beet plants grown in salt-affected soil and subjected to saline water irrigation during the 2020/2021 seasons. (**A**) Root yield (ton ha$^{-1}$), (**B**) Foliage yield (ton ha$^{-1}$), (**C**) Total yield (ton ha$^{-1}$), and (**D**) Sugar yield (ton ha$^{-1}$) during the 2020/2021 seasons, respectively. Data presented are the means ± standard deviation (mean±SD) of three biological replicates. Different letters signify statistically significant differences between treatments according to Tukey's HSD test ($p_{\text{Irrigation × Treatment}} \leq 0.05$).
